# CRISPR/Cas9-mediated efficient and heritable targeted mutagenesis in tomato plants in the first and later generations

**DOI:** 10.1038/srep24765

**Published:** 2016-04-21

**Authors:** Changtian Pan, Lei Ye, Li Qin, Xue Liu, Yanjun He, Jie Wang, Lifei Chen, Gang Lu

**Affiliations:** 1 Key Laboratory of Horticultural Plant Growth, Development and Quality Improvement, Ministry of Agriculture, Hangzhou 310058, China; 2 Zhejiang Provincial Key Laboratory of Horticultural Plant Integrative Biology, Department of Horticulture, Zhejiang University, Hangzhou 310058, China

## Abstract

The CRISPR/Cas9 system has successfully been used in various organisms for precise targeted gene editing. Although it has been demonstrated that CRISPR/Cas9 system can induce mutation in tomato plants, the stability of heredity in later generations and mutant specificity induced by the CRISPR/Cas9 system in tomato plants have not yet been elucidated in detail. In this study, two genes, *SlPDS* and *SlPIF4*, were used for testing targeted mutagenesis in tomato plants through an *Agrobacterium tumefaciens*-mediated transformation method. A high mutation frequency was observed in all tested targets in the T0 transgenic tomato plants, with an average frequency of 83.56%. Clear albino phenotypes were observed for the *psd* mutants. High frequencies of homozygous and biallelic mutants were detected even in T0 plants. The majority of the detected mutations were 1- to 3-nucleotide deletions, followed by 1-bp insertions. The target mutations in the T0 lines were stably transmitted to the T1 and T2 generations, without new modifications or revision. Off-target activities associated with *SlPDS* and *SlPIF4* were also evaluated by sequencing the putative off-target sites, and no clear off-target events were detected. Our results demonstrate that the CRISPR/Cas9 system is an efficient tool for generating stable and heritable modifications in tomato plants.

Precise genome editing provides great advantages in plant functional genomics research and crop improvement by generating tailored modifications at a target genome sequence, in contrast to traditional mutagenesis methods, such as physical and chemical mutagenesis, which are random and time-consuming. Three genome-editing tools have been developed, including zinc-finger nucleases (ZFNs), transcription activator-like effector nucleases (TALENs) and the clustered regularly interspaced palindromic repeat/CRISPR-associated protein 9 system (CRISPR/Cas9). These technologies induce site-specific double-strand breaks (DSBs) in a targeted fashion within the genome, generating modifications through homologous recombination (HR) or non-homologous end-joining (NHEJ) repair mechanisms^1^,^2^,^3^. HR can accurately repair DSBs using the homologous sequence flanking a DSB or an exogenously supplied DNA ‘donor template’ as the template. However, NHEJ repair is error-prone and frequently causes insertions or deletions (indels) around the sites of DNA breaks. In eukaryotic cells, DSBs are preferentially repaired by NHEJ events, thus providing a promising strategy for research on plant functional genomics and crop improvement^4^,^5^. In the past decade, ZFNs and TALENs have been successfully used in a variety of organisms^1^. However, the design and construction of sequence-specific DNA-binding modules are difficult and expensive^1^. Recently, a novel precise genome-editing tool, the CRISPR/Cas system, has been discovered, sparking a revolution in genome-editing fields. The CRISPR/Cas system exists extensively in many bacteria and most archaea as a defense system against invading genetic elements^6^. There are three CRISPR/Cas system types (I, II, and III), and the type II system originating from *Streptococcus pyogenes* can direct the cleavage of target DNA sites with a single Cas9 protein, thus making it promising for widely used in genome editing^7^,^8^. The CRISPR/Cas9 type II system is composed of three components: Cas9 endonuclease, CRISPR RNA (crRNA) and trans-activating crRNA (tracrRNA). The CRISPR/Cas9 protein is guided by a tracrRNA:crRNA duplex and cleaves the target DNA sequence complementary to the crRNA^1^. In the engineered system, the tracrRNA and crRNA complex has been fused into a single chimeric guide RNA (sgRNA) containing a 20-nucleotide (nt) sequence determining the DNA target sequence and the double-stranded structure required for Cas9 binding. The 3′-end of the target sequence adjoins with an NGG protospacer adjacent motif (PAM) that is recognized by the CRISPR/Cas9 system^8^. With its advantage of high efficiency, the CRISPR/Cas9 system has been widely used in eukaryotic cells since 2013^9^,^10^.

In plants, the CRISPR/Cas9 system has achieved effective genome editing in numerous species including *Arabidopsis thaliana*^11^,^12^,^13^, *Oryza sativa*^14^,^15^,^16^,^17^, *Nicotiana benthamiana*^18^,^19^,^20^, *Solanum lycopersicum*^21^, maize^22^, soybean^23^,^24^, sorghum^25^, *Citrus sinensis*^26^, *Triticum aestivum*^27^, *Marchantia polymorpha*^28^, and Populus^29^. The high mutation frequency of the CRISPR/Cas9 system has been proven in various plants through transient assays or genetic transformation methods. The mutation and inheritance patterns have been investigated in detail in Arabidopsis and rice^16^,^30^. In Arabidopsis, mutations induced by the CRISPR/Cas9 system occur mostly in somatic cells, thus resulting in a low frequency of homozygous mutation genotypes detected in T1 generation plants^12^,^17^,^30^. In contrast, in rice, homozygous and biallelic mutants are readily detected in T0 transgenic plants^17^. The inheritance of the targeted homozygous mutants is stable. The segregation pattern in the descendants of the heterozygous and biallelic mutants conforms to the classical Mendelian model^16^,^30^,^31^. Recently, the CRISPR/Cas9 system has been successfully used in crop improvement. In wheat, all three *MLO* homolog alleles have been knocked out by the CRISPR/Cas9 system, conferring broad-spectrum resistance to powdery mildew in wheat plants^27^.

The tomato (*S. lycopersicum* L.), an important economic crop, is regarded as an ideal model plant for studying plant reproductive development, functional genomics and quality improvement. It has been demonstrated that the CRISPR/Cas9 system can induce mutations in tomato plants by *Agrobacterium tumefaciens*-mediated transformation or transient assays in hairy roots with *A. rhizogens*^21^,^32^. The T0 mutations can be stably transmitted through the germ line^21^. However, the mutation types, inheritance pattern in later generations, and off-target activities of CRISPR/Cas9-induced mutations in tomato plants need to be systematically elucidated. Here, two tomato genes, phytoene desaturase (*SlPDS*, Solyc03g123760.2.1) and phytochrome interacting factor PIF4 (*SlPIF4*, Solyc07g043580.2.1), were targeted using computationally designed gRNAs with the stable transformed CRISPR/Cas9 system. *SlPDS* encodes phytoene desaturase, the key enzyme in carotenoid biosynthesis, and silencing the gene will cause photobleaching or albino phenotypes^33^. *SlPIF4*, a homologous gene of Arabidopsis *PIF4,* belongs to the basic helix-loop-helix multigene family^34^, a large superfamily of transcription factors, containing 159 members with highly conserved bHLH domains. It’s interesting to study the reliability of CRISPR/Cas9 system on multigene family function research^35^. The mutation and inheritance patterns of the target genes were calculated in the T0 and later generations by genotyping and sequencing. Off-target events were also evaluated. Our data showed that high rates of homozygous and biallelic mutants of *SlPIF4* were generated even in the first generation, using the CRISPR/Cas9 system. The gene modifications were stably transmitted to the T1 and T2 generations regardless of whether the T-DNA (transgene region) was present. These data demonstrate that the CRISPR/Cas9 system can efficiently and specifically induce heritable mutations in tomato plants.

## Results

### CRISPR/Cas9 system construction and sgRNA design

Two binary vectors were constructed to express Cas9 and sgRNA for gene editing. In both vectors, the Arabidopsis *U6-26* promoter was selected to generate sgRNA, whereas the expression of *Cas9* was driven by the CaMV 35S and *AtUBQ* promoter, respectively (Supplementary Figure S1a). Two genes, namely, *SlPDS* and *SlPIF4*, were selected as the targets of Cas9 endonuclease. Two independent sgRNAs following the PAM were designed for each gene (Supplementary Figure S1b). Therefore, a total of four binary vectors were constructed to evaluate the efficacy of the CRISPR/Cas9 system in inducing targeted mutagenesis in tomato plants.

### CRISPR/Cas9-mediated mutagenesis in transgenic tomato plants

**Figure 1:**
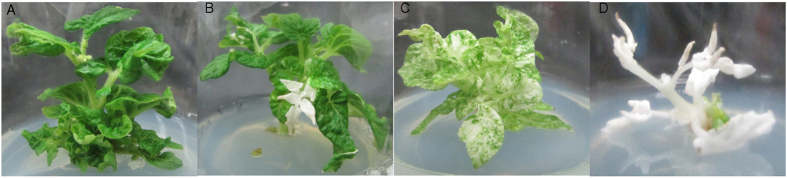
Albinism phenotype of *SlPDS* transgenic plants in T0 generation. (**A**) WT (contain T-DNA). (**B**,**C**) chimeric mutant. (**D**) biallelic mutant. Mutant B, C and D show albino phenotype to varying degrees.

Through the *A. tumefaciens*-mediated transformation method, 22, 7, 25 and 19 independent T0 transgenic lines were obtained in sgRNA1-*SlPDS*, sgRNA2-*SlPDS*, sgRNA1-*SlPIF4* and sgRNA2-*SlPIF4* constructs, respectively (Table 1). Remarkably, 54.54% (12 out of 22) of the sgRNA1-*SlPDS* and 57.14% (4 out of 7) of the sgRNA2-*SlPDS* transgenic plants showed an albino phenotype, indicating the complete or partial loss of *SlPDS* function. Some mutants showed an albino phenotype only in certain leaves, whereas in other mutants, all the leaves appeared to be albino to a variable extent, and some did not even survive to produce flowers and fruits because of their severe albino phenotype (Fig. 1). However, no obvious abnormal phenotype was observed in the *SlPIF4* transgenic positive plants.

**Figure 2:**
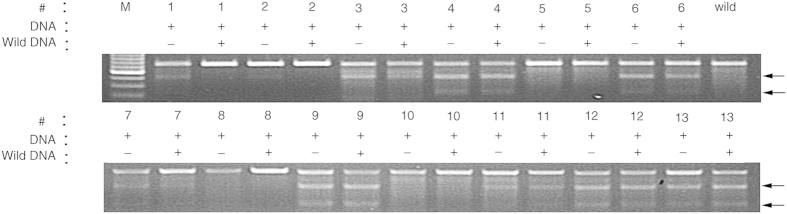
Detecting of target mutations by T7 endonuclease I (T7E1) assay. The target fragments were amplified by PCR from genomic DNA which was extracted from independent transgenic plants leaves. #1–13 represents 13 independent transgenic plants of sgRNA2-*SlPIF4.* Arrows indicate the digested fragments by T7E1. +: PCR products were added. −: no PCR products were added.

Mutations were first detected using the T7 endonuclease I (T7E1) assay at the target site with 13 randomly selected independent transgenic lines of sgRNA2-*SlPIF4* to estimate the efficiency of the CRISPR/Cas9 system (Fig. 2). The DNA fragments of target sites from 10 transgenic lines could be digested with T7E1 enzyme, and a high mutation rate of approximately 76.9% (10/13) occurred in the tested lines of sgRNA2-*SlPIF4.* The DNA fragments from sgRNA2-*SlPIF4* #5, #8 and #10 were not digested by T7E1, thus suggesting that those plants might be WT-type (containing T-DNA). To further verify the results, the target fragments of all 13 independent transgenic lines were sequenced, confirming that all these transgenic plants identified by T7E1 analysis contained mutant alleles at the target site. Meanwhile, the #8 and #10 lines, lacking detectable mutant alleles, were regarded as WT plants, consistently with the results of the T7E1 assay. However, #5 was homozygous, with one T base deletion at the 3rd base of the PAM (Supplementary Table S8). The minor discrepancy between the T7E1 and Sanger sequencing results indicated that the T7E1 method was more prone to errors due to experimental variations.

To accurately calculate the efficiency of the CRISPR/Cas9 system, the target sequences were analyzed by directly sequencing each transgenic plant of the four vectors. Of the 73 independent transgenic plants, 61 (83.56%) had mutations (Table 1). The mutation efficiencies were similar between two target sites in the same gene, i.e., the mutation rates were 84.00% and 89.47% for sgRNA1-*SlPIF4* and sgRNA2-*SlPIF4*, respectively (Table 1).

Interestingly, when comparing the editing efficiency of targets with different GC contents, we found that the three sgRNAs with GC content above 50% exhibited a high editing efficiency (84.00–100.00%), whereas the sgRNA1 of *SlPDS*, containing a relatively low GC content (40%), showed a lower editing efficiency (72.70%). Previous research has demonstrated that the GC content of sgRNA might influence its binding to its target site and ultimately its editing efficacy^36^.

### Genotyping of T0 independent transgenic lines

Tomato plants are diploid, and one or both copies of a gene may be cleaved when CRISPR/Cas9 is inserted into the genome, generating five genotypes in T0 transgenic lines, including homozygote, biallele, heterozygote, chimera, and WT-type^16^. To estimate the proportion of each genotype in the T0 mutants, a total of 72 T0 independent transgenic lines from sgRNA1/2-*SlPDS* and sgRNA1/2-*SlPIF4* were genotyped. For each plant, genomic DNA was extracted from tomato leaf samples, and 4–19 clones of the PCR amplicons were sequenced. Finally, more than 630 clones were selected and analyzed, and the genotype data are summarized in Table 2 (more details are in Supplementary Table S1–4).

Of 72 tested independent transgenic positive plants, 9 (12.5%) and 5 (6.9%) mutants were putative biallelic and homozygous, respectively (Table 2). Interestingly, sequence analysis showed that all homozygotes had single-base deletions, and most biallelic mutations were one- to three-base deletions (Supplementary Table S1–4). These results suggest that short deletions predominantly occurred in an early stage of embryonic cell division. Unexpectedly, there were no heterozygotes in all the examined T0 transgenic plants, which was quite different from previously reported results in rice^16^ and Arabidopsis^17^. In contrast, 46 (63.9%) T0 plants had chimeric mutations, in which at least two mutation types occurred in each, including deletions, insertions and combined mutations.

Notably, no modification was detected by a sequence analysis in 12 lines out of the 72 tested T0 positive plants. These non-mutated lines were designated as WT, even though they carried T-DNA. Using quantitative reverse transcription PCR (qRT-PCR) with specific primers (Supplementary Table S10), the expression levels of *Cas9* and sgRNA were examined to investigate the possible reasons for the failed editing. The expression levels of *Cas9* in positive plants were similar to those in WT, which were significantly higher than that of sgRNA (Supplementary Figure S2). The WT plants that failed in generating modifications in the target genes had very low levels of sgRNA, and nearly no sgRNA expression was detected in some WT plants. In contrast, the positive plants contained a relatively high sgRNA level, suggesting that the expression level of the target-sgRNA rather than *Cas9* might be the major limiting factor for genome editing in tomato plants, which is consistent with a recent report on Arabidopsis^17^.

To detect mutation types in different organs or tissues, tomato leaves, shoots and flower buds were collected and mixed from each of randomly selected 16 T0 plants. More than 10 clones of PCR amplicons of the target sequences were sequenced for each tested mixed tissue. There were no new mutations detected in the homozygotes, bialleles or WT types (Supplementary Table S5), indicating that the mutations of different parts induced by CRISPR/Cas9 came from the same source. However, in the chimeras, new mutation types were found in mutants containing WT alleles (Supplementary Table S5). The results further demonstrate that the sgRNA-Cas9 complex was continuously active when the WT copies existed^16^.

### Stability of regenerated plants in the T0 generation

After the first transgenic shoot was generated from embryogenic calli, the regeneration and propagation of transgenic plantlets were subjected to further analysis. Because the CRISPR/Cas9 system was active during the regeneration period, the stability of the gene modifications was analyzed by sequencing target fragments. The mutations were stably transmitted to the regenerated plants in all examined homozygotes, bialleles and WT types, without generating new mutation types (Supplementary Table S6). However, in chimeras, new mutation types were observed due to the existence of WT copies. For example, compared with the T0-9 of sgRNA2-*SlPIF4*, the regenerated plants T0-9 (1) generated two novel mutation types: 2-bp deletions (d2) and 1-bp insertions (i1).

### CRISPR/Cas9-induced mutation patterns

**Figure 3:**
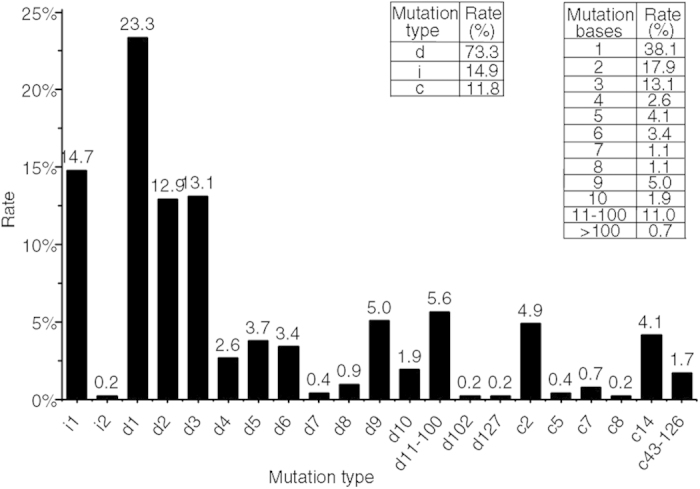
Pattern and frequency of CRISPR/Cas9-mediated mutations. The graph assembles the sequencing data of the four target sites in T0 and T1 transgenic plants. Left inset shows the frequency of insertion (i), deletion (d) and combined (c) mutation type. Right inset exhibits the occurrence rate of different mutation length. In x-axis: i#, number of bases insertion at target site; d#, number of bases deletion at target site; c#, combined mutations.

All the sequencing data collected from the four target sites were analyzed to further determine the mutation patterns and frequencies induced by the CRISPR/Cas9 system in tomato plants (Fig. 3, more details in Supplementary Table S8). A variety of targeted mutation types were observed in all detected clones, including deletions, insertions, and combined mutations. In 536 mutant clones, up to 73.3% (393/536) of mutations were deletion events, followed by insertion mutations (14.9%) and combined mutations (11.8%). CRISPR/Cas9-induced mutations were predominantly short nucleotide changes (≤3 bp) (approximately 70%), of which most (23.3%) involved the deletion of one nucleotide. All insertion mutations except for one clone were 1-bp insertions of A or T nucleotides, consistently with the results of a previous report^16^. Only 0.37% of mutations exhibited >100-bp deletions, and the largest deletion fragment had 127 bp. Previous reports have indicated that Cas9 usually cleaves target sites at the fourth base upstream of the PAM^37^. In our study, 63.0% of the mutations occurred at the 4th base of the PAM, and more than 18.4% occurred at the 5th base of the PAM in deletion events. However, almost all 1-bp deletions (92.8%) and 1-bp insertions (97.5%) occurred at the 4th base of the PAM.

### Inheritance and stability of mutations in T1 and T2 generations

The genotypes at the target sites of several T1 and T2 progeny were examined to investigate the transmission pattern of CRISPR/Cas9-mediated mutations (Table 3, Supplementary Table S7). For each T1 line, 8–22 progeny were selected and examined. As expected, all 14 and 15 T1 progeny of sgRNA1-*SlPIF4* T0-22 and sgRNA2-*SlPIF4* T0-19 homozygotes, respectively, were homozygous for the same mutations, indicating that the mutations in the homozygotes were stably passed to the next generation in a Mendelian fashion, which was consistent with previous reports^16^,^30^. For biallelic mutations, the normal segregation in progeny should conform to the segregation ratio of 1 (homozygous for mutation 1) : 2 (biallele) : 1 (homozygous for mutation 2)^30^,^38^. However, unexpected segregation ratios of 0:5:5 and 1:7:5 were observed in the T1 progeny of the sgRNA2-*SlPIF4* T0-8 and T0-10 lines, respectively. The results indicated that the two alleles in one biallelic mutant might not be inherited with equal frequencies^38^. The segregation patterns of the chimeras were less predictable, and a number of new mutants were found in the T1 lines. Homozygotes were readily detected in the T1 progeny of the examined chimeras. For example, 10/12 (83%) progeny of sgRNA2-*SlPIF4* T0-12 were homozygous. As expected, the progeny of the WT T0 plants were not detected to have any targeted mutations (Table 3).

The presence of the transgene region (T-DNA) was examined in T1 lines. The T-DNA could be segregated out in the progeny of most T0 lines, with 90% of the T0 lines generating T-DNA-free progeny. However, on average, 26% (36/138) of the T1 plants were detected to be T-DNA-free mutants (Table 3). To further investigate the genetic stability of the targeted mutations of the T-DNA-free mutants, the genotypes of a total of 54 T2 plants derived from randomly selected T1 T-DNA-free homozygotes were analyzed in detail (Supplementary Table S7). All the descendants were T-DNA-free plants and showed the same target mutations as the T1 plants, without any new mutations or revisions. The result indicated that targeted mutations could be stably passed to later generations in T-DNA-free plants, thus providing an effective strategy for tomato improvement.

### Off-target activity in tomato plants

In plants, low-frequency cases of off-target cleavage have been reported^14^,^16^,^24^,^39^. To detect the off-target events in tomato plants, potential off-target loci following a PAM sequence and highly homologous to *SlPDS* and *SlPIF4* target sites were predicted with the website tool CRISPR-P^40^. The three most likely off-target sites of each target for sgRNA1-*SlPDS* and sgRNA1/2-*SlPIF4* were selected and examined in 30 randomly selected T0 and T1 plants (Table 4). Previous reports have indicated that the 12 nucleotides located at the target site and adjoining the PAM, as a “seed sequence”, are very important for specific recognition and efficient target cleavage for Cas9^41^,^42^,^43^. In our chosen candidates, 1 to 3 mismatch bases existed in the ‘seed sequence’. Genomic DNA was extracted from the leaves of each tested transgenic plant, and then the putative off-target sites were amplified using PCR with specific primers (Supplementary Table S10). No mutations were found in the putative off-target sites in all tested T0 and T1 transgenic plants, thus indicating that CRISPR/Cas9 system-induced mutagenesis is highly specific in tomato plants.

## Discussion

Tomatoes are one of the most important crops worldwide and are the second most consumed vegetable in the world. Because of the availability of its entire genome sequence and its well-studied genomics, the tomato is regarded as an important model plant for studying flower and fruit development^44^. Screening for targeted mutants is a useful strategy for researching plant functional genomics and crop improvement. However, the screening process is exceedingly laborious and time-consuming through traditional mutagenesis methods. Using genome-editing technology, precise, efficient and simple site-directed mutagenesis has been achieved in plants. Since the first successful application of the CRISPR/Cas9 system in the zebrafish, the CRISPR/Cas9 system has widely been used for genome editing in animals and plants because of its simplicity, efficiency and versatility^11^,^12^,^13^,^14^,^15^,^16^,^17^,^18^,^19^,^20^,^21^,^22^,^23^,^24^,^25^,^26^,^27^,^28^. Previous studies have proven that the CRISPR/Cas9 system can induce mutations in tomato plants^21^,^32^. In this study, to systematically elucidate the application of the CRISPR/Cas9 system in tomato plants, two different genes were selected and targeted by using a previously reported CRISPR/Cas9 system^13^. We provide the comparative data on mutation efficiencies, mutation types, and hereditary stability in the T1 and T2 generations and cleavage specificity in tomato plants. Our results showed that the gRNA:Cas9-induced mutation rate was 83.56% on average in the T0 transgenic tomato plants (Table 1). Considering the homologous recombination-based repair (HR), the mutation efficiency of CRISPR/Cas9 would be higher. Previous reports have shown that the utilization of plant endogenous promoters to express *Cas9* generates higher mutation efficiencies than CaMV 35S in monocotyledons^14^,^15^,^22^,^25^,^45^. In dicotyledons, the mutation frequency varies within a large range from 26 ~ 95% when the CaMV 35S promoter is used^13^,^20^,^46^. Our data indicated that the Arabidopsis *UBQ* and 2 × *CaMV35S* promoters driving the Cas9 endonuclease (Supplementary Figure S1) efficiently induced DSB in the target sites with similar mutation efficiency. However, in soybean and liverwort, the mutation efficiencies of CRISPR/Cas9 can increase 2–7-fold when their intrinsic *U6* promoter is used instead of the Arabidopsis *U6* promoter^24^,^28^. Through comparison with the sequences of Arabidopsis *U6* small nuclear RNA (snRNA), 7 *SlU6* genes were identified in the tomato genome (Supplementary Figure S3 and Table S9), all containing two highly conserved elements: an upstream sequence element (USE, consensus sequence RTCCCACATCG) and a TATA-like box, which indicates that the tomato *U6* promoters may have similar functions to the Arabidopsis *U6* promoters. Recent studies have shown that using the tissue-specific *gata1* promoter in the zebrafish imparts major advantages in the study of tissue-specific genes, and the application of a germ-line-specific *SP* (*SPOROCYTELESS*) promoter in Arabidopsis generates a high proportion of homozygous mutants^47^,^48^. Therefore, the choice of promoters to drive sRNA or Cas9 expression depends on the experimental objectives and target genes. The GC content of the target sites also influenced mutation efficiency: GC content over 50% generated high editing efficiencies (91.16%) and lower GC content (40%) resulted in low editing efficiency (72.7%). Similar phenomena have been reported in Arabidopsis and rice^16^,^30^,^43^. However, higher GC contents of the specific targeted sequence might increase the off-target activity^49^.

High percentages of homozygous (11.36%) and biallelic (18.18%) mutants were observed in *SlPIF4* T0 transgenic plants (Table 2). Only one biallele was found among 29 independent *SlPDS* transgenic plants, which might be attributed to the severe albino phenotype, because some plants could not survive when the *SlPDS* gene was completely knocked out. Unexpectedly, there were no heterozygote among the T0 transgenic tomato plants, a result quite different from those in rice and Arabidopsis^16^,^17^. Instead, we observed a high frequency of somatic mutations in T0 transgenic plants, with 63.9% (46/72) of mutants being chimeric. We speculate that the sgRNA-Cas9 complex may be more active in tomato plants than in Arabidopsis and rice, and that this activity may have facilitated transformation of heterozygotes into chimeras in the early growth stages of our T0 plants, through the continuous modification of the WT alleles in the heterozygotes by the sgRNA-Cas9 complex. Similar modification events have also been reported in Arabidopsis, rice, and soybeans^16^,^17^,^30^. New mutations were detected in different tissues or organs, and regenerated plantlets from chimeras containing WT alleles also supported the above assumption (Supplementary Tables S5 and S6). For homozygotes and bialleles, gene modifications can be stably maintained during plant regeneration from T0 transgenic plants. It is useful for function studies to obtain sufficient amounts of uniform transgenic plants by regeneration from embryonic cells in the T0 generation. The varieties of mutations were created by NHEJ in T0 plants. They were mostly 1- to 3-bp deletions (49.2%), followed by 1-bp insertions (14.7%), and 1-bp deletions occurred preferentially (23.3%) in deletion events (Fig. 3). However, 1-bp insertions predominantly occur in Arabidopsis and rice^16^,^30^. This discrepancy may be caused by the difference in intrinsic DNA-repair mechanisms among different plant species^16^. By comparing the genotypes of T0, T1, and T2 generation plants (Table 3, Supplementary Table S7), it is clear that the mutations of the homozygotes and bialleles were stably passed on to later generations regardless of whether the T-DNA (transgene region) was present.

Off-target events are a common concern in the application of the CRISPR/Cas9 system in plants. Previous reports have indicated that the off-target activity of the CRISPR/Cas9 system varies among organisms, being high in humans and low in mice and zebrafish^50^,^51^,^52^,^53^. In plants, low-probability off-target events are found in rice and soybeans due to the specificity of the sgRNA design^16^,^24^,^38^. In our study, no off-target events were detected in candidates of off-target sites by sequence assay in the T0 and T1 generations (Table 4), suggesting the high specificity of the CRISPR/Cas9 system in tomato plants. Many factors affect the Cas9/sgRNA targeting specificity. The PAM-proximal region of the sgRNA guiding sequence is the most important factor for determining the binding specificity^54^. Hence, designing a highly specific target sequence is the most effective strategy to reduce off-target events. Several bioinformatic tools have been developed that can provide highly specific sgRNAs for model and crop plants^40^,^55^. Off-targeting can be reduced by 50- to 1500-fold by using a double nicking strategy mediated by a pair of Cas9 nickase (Cas9n) enzymes with sgRNA^56^. The specificity of Cas9 also depends on the relative abundance of the effective Cas9/sgRNA complex with respect to the effective target concentration^49^. Compared with Cas9 endonuclease, the latest CRISPR-Cpf1 system can recognize different PAMs and can be efficiently targeted, thus providing a replacement strategy to reduce off-target activity^57^.

In this study, the CRISPR/Cas9 system efficiently induced DSB for *SlPDS* and *SlPIF4* at four target sites in tomato plants. Homozygous and biallelic mutants were readily found in the T0 generation, and the mutations were stably transmitted to the T1 and T2 generations regardless of whether the T-DNA (transgene region) was present, without new mutations or reversions, thus providing an effective strategy for tomato improvement. No off-target events were detected in the putative off-target sites in T0 and T1 generation plants, indicating that the CRISPR/Cas9 system is highly specific in the tomato. In short, as a powerful editing tool, the CRISPR/Cas9 system will accelerate basic research and genetic improvement in tomato plants.

## Methods

### Vector construction

Three backbones of AtU6-sgRNA, hSpCas9 and psgR-Cas9 were obtained from Prof. Jian-Kang Zhu (Chinese Academy of Sciences, Shanghai, China). Here, two forms of the pCAMBIA1301 binary vector were constructed: AtU6-sgRNA-2 × 35S-Cas9 and AtU6-sgRNA-AtUBQ-Cas9. The target sequences were designed using a web tool of CRISPR-P^40^. For constructing AtU6-sgRNA-2 × 35S-Cas9, the synthesized oligos were annealed and inserted into *Bbs* I sites of the AtU6-sgRNA vector according to procotol^58^. Then, the fragment of AtU6-sgRNA was fused to Cas9 digested by *Kpn* I and *Sal* I and inserted into the *Kpn* I/*Xba* I sites of the pCAMBIA1301 binary vector. For constructing the AtU6-sgRNA-AtUBQ-Cas9 vector, the synthesized sequences were annealed and inserted into *Bbs* I sites of the AtU6-sgRNA-AtUBQ-Cas9 vector, and the AtU6-sgRNA-AtUBQ-Cas9 cassette was inserted into the *Kpn* I/*Hind* III sites of the pCAMBIA1301 binary vector.

### Growth and transformation of tomato plants

The tomato cultivar ‘Micro-Tom’, provided by the Tomato Genetics Resource Center (University of California, Davis), was used for *A. tumefaciens*-mediated transformation. The pCAMBIA1301 vectors containing the sgRNA and Cas9 expression cassette were transformed into *Agrobacterium* strain GV3101 by the freeze-thaw method. The binary vectors were transformed into tomato plants through the leaf-disc method^59^. In brief, tomato seeds were germinated on 1/2 MSO medium after sterilization with 10% NaClO. After 6–8 days culture, the intermediate cotyledons were excised into small slices of approximately 1 cm and then transformed with *Agrobacterium*. The explants were inoculated on selective plates with hygromycin (6 mg/L) until transgenic plants were regenerated from the calluses. After rooting, the regenerated transgenic plants were moved to a light growth chamber with a 16-h-light (25 ± 1 °C)/8-h-dark (20 ± 1 °C) photoperiod.

### T7 Endonuclease I (T7EI) assay and genotyping

Genomic DNA from tomato T0 transgenic plants was extracted using CTAB methods, and the genomic flanks containing the target sites were amplified using specific primers (Supplementary Table S10). Then, 300 ng of purified PCR products were denatured-annealed and digested with T7EI enzyme (NEB, USA) at 95 °C for 5 minutes in a water bath and were then allowed to cool to room temperature. The annealed PCR products were digested with 0.5 μl T7EI for 1 h at 37 °C and then were subjected to 2% agarose gel electrophoresis. For genotyping of T0 plant, the PCR products amplified with specific primers (Supplementary Table S10) were directly cloned into the pGEM-T easy Vector (Promega, USA), and approximately 10 clones were sequenced for each plant with the M13 primer. For the genotyping of T1 and T2 plants obtained from the T0 lines by strict self-pollination, the target fragments were directly sequenced.

### Off-target analysis

The potential off-target sites of the target sequence were predicted with the web tool of CRISPR-P^40^ using the full 20-bp target sequence for the Blastn algorithm. These top-ranking potential off-target sites containing fewer than 3-bp mismatches in the 12-bp seed sequence were selected. The genomic DNA surrounding the potential off-target sites was amplified using specific primers (Supplementary Table S10). PCR products were analyzed by Sanger sequencing.

### Quantitative RT-PCR

Total RNA was extracted from T0 transgenic plant leaves using a Total RNA Kit II (OMEGA, USA). One microgram of RNA was used to synthesize the first cDNA using the PrimeScript^TM^ RT reagent kit (Takara, Japan) according to the manufacturer’s protocol. qRT-PCR was performed in a volume of 15 μl on a CFX96 Real-Time system (Bio-Rad) with *Cas9* and sgRNAs specific primers (Supplementary Table S10). The *SlUbi3* (GenBank accession number X58253) was amplified synchronously as an internal control. The expression level was calculated using the 2^−△△CT^ method.

## Additional Information

**How to cite this article**: Pan, C. *et al.* CRISPR/Cas9-mediated efficient and heritable targeted mutagenesis in tomato plants in the first and later generations. *Sci. Rep.* **6**, 24765; doi: 10.1038/srep24765 (2016).

## Supplementary Material

Supplementary Information

## Figures and Tables

**Table 1:** Percentage of T0 transgenic plants examined with mutations and GC content of sgRNAs.

Vector	Target gene	sgRNA	No. of lines	No. of lines with mutations	Mutation rate	sgRNA GC content
AtU6-sgRNA-AtUBQ-Cas9	*SlPDS*	sgRNA1	22	16	72.70%	40.00%
	*SlPDS*	sgRNA2	7	7	100.00%	55.00%
AtU6-sgRNA-2 × CaMV 35S-Cas9	*SlPIF4*	sgRNA1	25	21	84.00%	60.00%
	*SlPIF4*	sgRNA2	19	17	89.47%	50.00%
Total	2	4	73	61	83.56%	51.25%

**Table 2:** Detected zygosity of T0 independent transgenic lines of sg1/2-*SlPDS* and sg1/2-*SlPIF4*.

Target gene	sites	No. of examed lines	Zygosity$			
			Homozygote	Biallele	Chimera	WT#
*SlPDS*	sgRNA1	21	0	0	15(71.4%)	6(28.6%)
*SlPDS*	sgRNA2	7	0	1 (14.3%)	6 (85.7%)	0
*SlPIF4*	sgRNA1	25	2 (8.0%)	3 (12.0%)	16 (60.0%)	4 (16.0%)
*SlPIF4*	sgRNA2	19	3 (15.8%)	5 (26.3%)	9 (47.4%)	2 (10.5%)
Total		72	5 (6.9%)	9 (12.5%)	46 (63.9%)	12 (16.7%)

^$^The zygosoty of homozygote, biallele and chimera in T0 plant lines were putative.

^#^WT, wild-type sequence without mutations detected at target sites.

**Table 3:** Segregation patterns of CRISPR/Cas9-medicated targeted mutagenesis during the T0 to T1 generation.

Target gene	sgRNA	Line#	T0		T1	
			Zygosity$	Genotype	Mutation segregation	T-DNA
*SlPIF4*	1	T0-22	Homozygote	d1d1	15d1d1	12+;3−
*SlPIF4*	2	T0-19	Homozygote	d1d1	14d1d1	10+:4−
*SlPIF4*	2	T0-8	Biallele	d2,d9	3d2d2,6e*,5d9d9	10+:4−
*SlPIF4*	2	T0-10	Biallele	d3,i1	1d3d3,7e,5i1i1	8+:5−
*SlPDS*	1	T0-20	Chimera	d5,d12,c102,WT	8e	5+;1−
*SlPIF4*	1	T0-3	Chimera	d3,d4,i1	13e,2i1	11+:4−
*SlPIF4*	2	T0-12	Chimera	d2,d6,d17,c7	5d6,2e,5d2	All+
*SlPIF4*	2	T0-16	Chimera	d1,d2,d3	8d2,11e,3d1	14+:8−
*SlPIF4*	1	T0-6	WT	WT	13WT	12+:1−
*SlPDS*	1	T0-18	WT	WT	16WT	9+:7−

^#^Line name is in the format of T0-#.

^$^The zygosoty of homozygote, biallele and chimera in T0 plant lines were putative. d#, # of bp deleted at the target sites; i#, # number of bases insertion at target sites; c#, combined mutation; WT, wild-type sequence without mutations detected at target sites.

^*^e, heterogeneous, more than one sequence detected in the sample; +, T-DNA was detected; −, T-DNA was not detected.

**Table 4:**
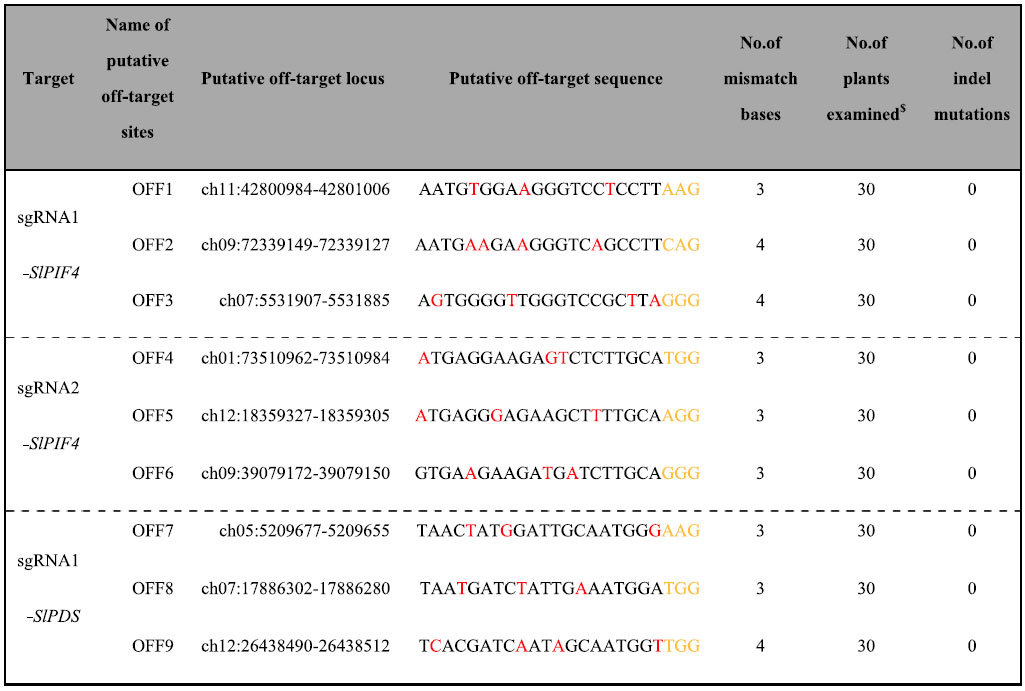
Mutation analyzed of potential off-target sites.

PAM sequence (NGG) is indicated in orange, the analogue NAG is also used for testing. Mismatch nucleotides are marked in red. ^$^Examined plants were randomly selected from the T0 and T1 generations of sgRNA1/2-*SlPIF4* and sgRNA1-*SlPDS*, with the T1 plants were Cas9 positive or negative.
