# Corrigendum: CRISPR/Cas9-mediated efficient and heritable targeted mutagenesis in tomato plants in the first and later generations

**DOI:** 10.1038/srep46916

**Published:** 2017-12-22

**Authors:** Changtian Pan, Lei Ye, Li Qin, Xue Liu, Yanjun He, Jie Wang, Lifei Chen, Gang Lu

Scientific Reports 6: Article number: 24765; 10.1038/srep24765 published online: 04 21 2016; updated: 12 22 2017.

The original version of this Article contained an error in Affiliation 1, which was incorrectly given as ‘Key Laboratory of Horticultural Plant Growth, Development and Quality Improvement, Ministry of Agriculture, Hangzhou 310058, China’. The correct affiliation is listed below:

Key Laboratory of Horticultural Plant Growth, Development and Quality Improvement, Ministry of Agriculture, Zhejiang University, Hangzhou, 310058, China

This error has now been corrected in the PDF and HTML versions of the Article.

