# Supplementary material for: CRISPR/Cas9-mediated efficient and heritable targeted mutagenesis in tomato plants in the first and later generations
Source: Sci Rep. 2016 Apr 21;6:24765. doi: 10.1038/srep24765 (PMC4838866; doi:10.1038/srep24765)
Supplement: Supplementary Information [file srep24765-s1.pdf]

# CRISPR/Cas9-mediated efficient and heritable targeted mutagenesis in tomato plants in the first and later generations

Changtian Pan[1,2], Lei Ye[1,2], Li Qin [2], Xue Liu[2], Yanjun He[2], Jie wang[2], Lifei Chen[2], Gang Lu [1,2]*

## Supplementary Information

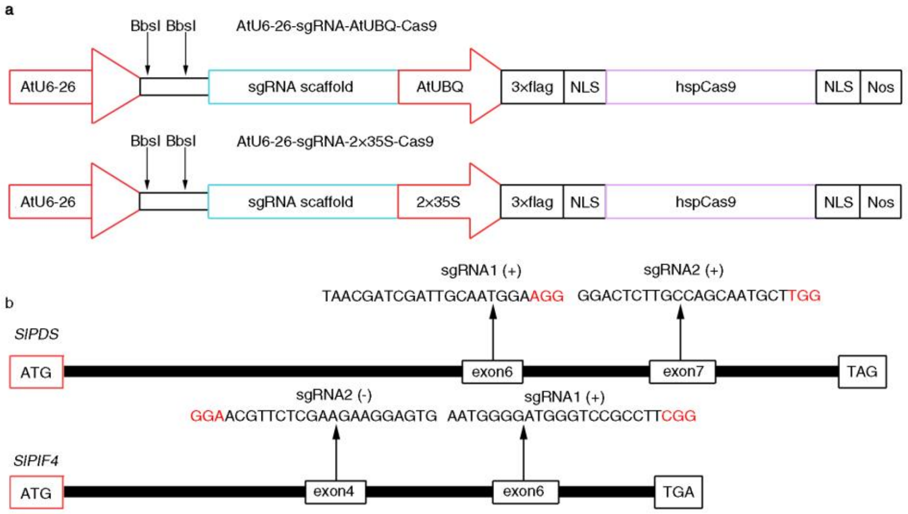


**Figure S1. Schematic diagrams for binary vectors used in this study and selected targets sites in *SlPDS* and *SlPIF4*.** (**a**) Schematic view of the expression cassettes. Cas9 fused with a flag tag (3×flag) and two nuclear localization signal (NLS) is expressed by either *Cauliflower mosaic virus* 35S promoter or *Arabidopsis* UBQ promoter. The scaffold sequence of sgRNA is derived using U6 promoters of *Arabidopsis*. Two *Bbs* I sites are located among the U6 promoter and sgRNA scaffold. (**b**) Schematic illustrating the target sites of *SlPDS* and *SlPIF4*. All the 20-bp sequence are followed by a PAM sequence (NGG; red). +: plus strand; -: minus strand. ATG: initiation codon; TAG or TGA: termination codon.

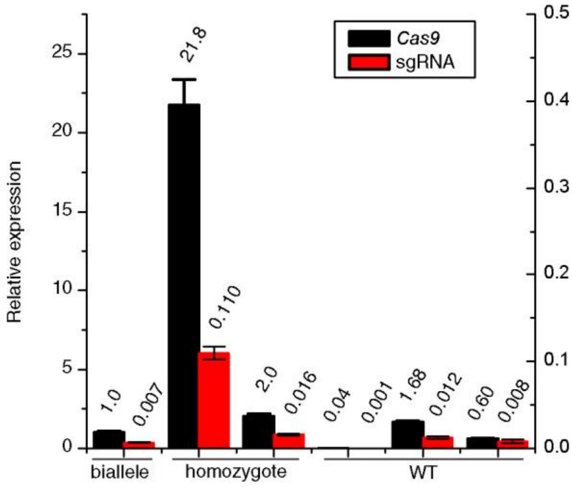


**Figure S2. Expression analysis of *Cas9* and sgRNA by RT–qPCR.** #WT, wild-type sequence with no mutation detected. $The zygosoty of homozygote, biallele and WT in T0 lines are putative. The *SlUbi3* was used as the internal control. The RT–qPCR products were verified by sequencing.

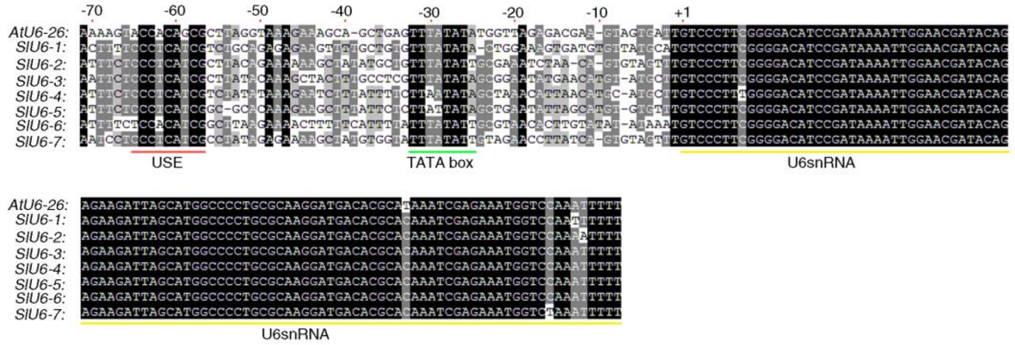


**Figure S3. Alignment of 7 tomato *U6* small nuclear RNA genes with Arabidopsis *U6-26* genes.** Tomato *U6* and Arabidopsis *U6-26* are highly-conserved. +1; transcription start site of U6 snRNA. Upstream sequence element (USE), TATA-like elements and U6 small nuclear RNA (snRNA) sequence regions are underlined.

**Supplementary Table S1.** Genotyping of T0 independent transgenic lines of sgRNA1-*SlPDS*

| Target | Lines# | Zygosity$ | Genotypes | Total | Ratio |
|---|---|---|---|---|---|
| sgRNA1-*SlPDS* | T0-1 | Chimera | 1d1,6i1,2WT | 15 | 71.4% |
| | T0-2 | Chimera | 8d1,1d8,1d28,1i1,2WT | | |
| | T0-4 | Chimera | 1d28,1c14,3WT | | |
| | T0-5 | Chimera | 1d2,6i1,3WT | | |
| | T0-6 | Chimera | 1d6,1i1,1c8,7WT | | |
| | T0-10 | Chimera | 2d1,1d65,3i1,4WT | | |
| | T0-11 | Chimera | 1d1,1d1a,2i1,3WT | | |
| | T0-12 | Chimera | 1d2,3d72,3WT | | |
| | T0-14 | Chimera | 1d55,2i1,2i1b,3WT | | |
| | T0-15 | Chimera | 1d1,1i1,4WT | | |
| | T0-16 | Chimera | 3d1,6i1,1i1a, | | |
| | T0-19 | Chimera | 5d5,2d11,1i1,3WT | | |
| | T0-20 | Chimera | 2d5,1d12,1c102,6WT | | |
| | T0-21 | Chimera | 1d2,1d4,2i1 | | |
| | T0-22 | Chimera | 1d2,7i1,2WT | | |
| | T0-3 | WT* | | 6 | 28.6% |
| | T0-7 | WT | | | |
| | T0-8 | WT | | | |
| | T0-9 | WT | | | |
| | T0-13 | WT | | | |
| | T0-18 | WT | | | |

#Line name is in the format of T0-#. $The zygosoty of T0 lines is putative. *WT, no mutation detected at target sites. d#, # of bp deleted at the target sites; i#, # of bases insertion at target sites; i#a, the same number of insertions at one site; i#b, the same number of insertions at other sites; c#, combined mutation.

**Supplementary Table S2.** Genotyping of T0 independent transgenic lines of sgRNA2-*SlPDS*

| Target | Lines | Zygosity | Genotypes | Total | Ratio |
|---|---|---|---|---|---|
| sgRNA2-*SlPDS* | T0-5 | Biallele | 14d3,5d5 | 1 | 14.3% |
| | T0-1 | Chimera | 1d2,1d4,1d23,1i2,1WT | 6 | 85.8% |
| | T0-2 | Chimera | 1d2,3d5,1i1 | | |
| | T0-3 | Chimera | 3d1,1d5,1WT | | |
| | T0-4 | Chimera | 2d1,1d3,1d4,1d102 | | |
| | T0-6 | Chimera | 1d1,2d3,3WT | | |
| | T0-7 | Chimera | 6d3,1d5,4d14 | | |

See Supplementary Table S1 for lines naming and genotype denotation.

**Supplementary Table S3.** Genotyping of T0 independent transgenic lines of sgRNA1-*SlPIF4*.

| Target | Lines | Zygosity | Genotypes | Total | Ratio |
|---|---|---|---|---|---|
| sgRNA1-*SlPIF4* | T0-22 | Homozygote | d1 | 2 | 8.0% |
| | T0-23 | Homozygote | d1 | | |
| | T0-7 | Biallele | 7c2,7c14 | 3 | 18.8% |
| | T0-15 | Biallele | 8d1,4d3 | | |
| | T0-19 | Biallele | 9d1,1d19 | | |
| | T0-1 | Chimera | 1d1,1d6,2WT | 16 | 64.0% |
| | T0-2 | Chimera | 2d4,2d18,5WT | | |
| | T0-3 | Chimera | 3d3,1d4,5i1 | | |
| | T0-4 | Chimera | 2d1,1d20,1d61 | | |
| | T0-5 | Chimera | 1d3,1d30,2WT | | |
| | T0-8 | Chimera | 1d1,1d3,1d7,1WT | | |
| | T0-9 | Chimera | 2d1,2d18,1c5 | | |
| | T0-10 | Chimera | 2c51,1c126,1WT | | |
| | T0-11 | Chimera | 4d8,1c51,1c52,3WT | | |
| | T0-13 | Chimera | 2d1,1d4,1d7 | | |
| | T0-14 | Chimera | 2d1,2d4,1WT | | |
| | T0-16 | Chimera | 2d1,5d3,1d5,1i1 | | |
| | T0-20 | Chimera | 1d1,1d5,2WT | | |
| | T0-21 | Chimera | 4d1,1d4,1d50,3i1 | | |
| | T0-24 | Chimera | 2d1,1d4,1WT | | |
| | T0-25 | Chimera | 5d1,1d4,1d5 | | |
| | T0-6 | WT | | 4 | 16.0% |
| | T0-12 | WT | | | |
| | T0-17 | WT | | | |
| | T0-18 | WT | | | |

See Supplementary Table S1 for lines naming and genotype denotation.

**Supplementary Table S4.** Genotyping of T0 independent transgenic lines of sgRNA2-*SlPIF4*.

| Target | Lines | Zygosity | Genotypes | Total | Ratio |
|---|---|---|---|---|---|
| sgRNA2-*SlPIF4* | T0-13 | Homozygote | d1 | 3 | 15.8% |
| | T0-17 | Homozygote | d1 | | |
| | T0-19 | Homozygote | d1 | | |
| | T0-2 | Biallele | 13d1,2d3 | 5 | 26.3% |
| | T0-7 | Biallele | 9d2,4d9 | | |
| | T0-8 | Biallele | 6d2,6d9 | | |
| | T0-10 | Biallele | 6d3,7i1 | | |
| | T0-18 | Biallele | 7i1,6c43 | | |
| | T0-1 | Chimera | 1d127,5i1,1WT | 9 | 47.4% |
| | T0-4 | Chimera | 1d1,4d6,3d17 | | |
| | T0-6 | Chimera | 3d2,2d2a,2d3,1d4,2d9 | | |
| | T0-9 | Chimera | 3d1,5d3,2d6 | | |
| | T0-11 | Chimera | 3d2,1d3,2i1 | | |
| | T0-12 | Chimera | 3d2,1d6,1d17,4d6a | | |
| | T0-14 | Chimera | 2d1,3d6,1d17,2i1 | | |
| | T0-15 | Chimera | 5d1,4d2,1d2a | | |
| | T0-16 | Chimera | 3d1,1d2,1d3 | | |
| | T0-3 | WT | | 2 | 10.5% |
| | T0-5 | WT | | | |

See Supplementary Table S1 for lines naming and genotype denotation. d#a, the same number bases of deletion at one site.

**Supplementary Table S5.** Compare the genotypes of leaves and mixed tissues.

| Target | Lines | Zygosity | Genotypes of leaf | Genotypes of mixed tissues* |
|---|---|---|---|---|
| sgRNA1-*SlPIF4* | T0-22 | Homozygote | d1 | d1 |
| sgRNA1-*SlPIF4* | T0-23 | Homozygote | d1 | d1 |
| sgRNA2-*SlPIF4* | T0-13 | Homozygote | d1 | d1 |
| sgRNA2-*SlPIF4* | T0-17 | Homozygote | d1 | d1 |
| sgRNA2-*SlPIF4* | T0-19 | Homozygote | d1 | d1 |
| sgRNA1-*SlPIF4* | T0-7 | Biallele | 7c2,7c14 | 4c2,6c14 |
| sgRNA1-*SlPIF4* | T0-15 | Biallele | 8d1,4d3 | 5d1,4d3 |
| sgRNA2-*SlPIF4* | T0-7 | Biallele | 9d2,4d9 | 5d2,2d9 |
| sgRNA2-*SlPIF4* | T0-8 | Biallele | 6d2,6d9 | 6d2,2d9 |
| sgRNA2-*SlPIF4* | T0-9 | Chimera | 3d1,5d3,2d6 | 2d1,5d3,3d6 |
| sgRNA1-*SlPDS* | T0-22 | Chimera | 1d2,7i1,2WT | <u>1d6</u>,<u>1d13</u>,2i1,6WT |
| sgRNA2-*SlPIF4* | T0-15 | Chimera | 5d1,4d2,1c3 | 8d1,8d2 |
| sgRNA1-*SlPIF4* | T0-6 | WT | N# | N |
| sgRNA2-*SlPIF4* | T0-5 | WT | N | N |
| sgRNA1-*SlPDS* | T0-13 | WT | N | N |
| sgRNA1-*SlPDS* | T0-18 | WT | N | N |

See Supplementary Table S1 for lines naming and genotype denotation. *Mixed tissues included leaves, shoots and flowers; #N, no mutation detected. Underline means new mutation were detected.

**Supplementary Table S6.** Genotyping the regeneration plantlets in T0 generation.

| Target | Lines[#] | Zygosity | Genotypes |
|---|---|---|---|
| sgRNA2-*SlPIF4* | T0-13 | Homozygote | d1 |
| sgRNA2-*SlPIF4* | T0-13(1) | Homozygote | d1 |
| sgRNA2-*SlPIF4* | T0-13(2) | Homozygote | d1 |
| sgRNA2-*SlPIF4* | T0-17 | Homozygote | d1 |
| sgRNA2-*SlPIF4* | T0-17(1) | Homozygote | d1 |
| sgRNA2-*SlPIF4* | T0-17(2) | Homozygote | d1 |
| sgRNA1-*SlPIF4* | T0-22 | Homozygote | d1 |
| sgRNA1-*SlPIF4* | T0-22(1) | Homozygote | d1 |
| sgRNA1-*SlPIF4* | T0-22(2) | Homozygote | d1 |
| sgRNA1-*SlPIF4* | T0-7 | Biallele | 7c2,5c14 |
| sgRNA1-*SlPIF4* | T0-7(1) | Biallele | 8c2,3c14 |
| sgRNA2-*SlPIF4* | T0-7 | Biallele | 9d2,4d9 |
| sgRNA2-*SlPIF4* | T0-7(1) | Biallele | 6d2,3d9 |
| sgRNA2-*SlPIF4* | T0-7(2) | Biallele | 4d2,4d9 |
| sgRNA1-*SlPIF4* | T0-1 | Chimera | 1d1,1d6,2WT |
| sgRNA1-*SlPIF4* | T0-1(1) | Chimera | 2d1,4d10,3WT |
| sgRNA1-*SlPIF4* | T0-1(2) | Chimera | 1d1,6d10,3WT |
| sgRNA2-*SlPIF4* | T0-9 | Chimera | 3d1,5d3,2d6 |
| sgRNA2-*SlPIF4* | T0-9(1) | Chimera | 1d2,2d3,5i1,2WT |
| sgRNA2-*SlPIF4* | T0-9(2) | Chimera | 1d1,1i1,7WT |
| sgRNA1-*SlPIF4* | T0-6 | WT | WT |
| sgRNA1-*SlPIF4* | T0-6(1) | WT | WT |
| sgRNA1-*SlPIF4* | T0-6(2) | WT | WT |
| sgRNA2-*SlPIF4* | T0-5 | WT | WT |
| sgRNA2-*SlPIF4* | T0-5(1) | WT | WT |
| sgRNA2-*SlPIF4* | T0-5(2) | WT | WT |

[#]Line name of regeneration plantlets is in the format of T0-M(N), with N designated the plantlets regenerated from the T0 plant T0-M. Underline means new mutation were detected.

**Supplementary Table S7.** Segregation patterns of CRISPR/Cas9-medicated targeted mutagenesis during the T1 to T2 generation in T-DNA-free plants.

| Target gene | sgRNA | Line# | T1 | | | T2 | |
|---|---|---|---|---|---|---|---|
| | | | Zygosity | Genotype | T-DNA | Mutation segregation | T-DNA |
| *SlPIF4* | 1 | T1-22(14) | Homozygote | d1d1 | - | 15d1d1 | 15- |
| *SlPIF4* | 1 | T1-8(10) | Homozygote | d1d1 | - | 17d1d1 | 17- |
| *SlPIF4* | 2 | T1-16(3) | Homozygote | d2d2 | - | 10d2d2 | 10- |
| *SlPIF4* | 2 | T1-12(7) | Homozygote | d2d2 | - | 12d2d2 | 12- |

#Line name is in the format of T1-M(N), with N designated the progeny of T1 plant T1-M. −, T-DNA was not detected.

**Supplementary Table S8.** Sequencing results of sgRNA1/2-*SlPDS* and sgRNA1/2-*SlPIF4* in T0 plants.

| Target | Genotypes | The sequences of target sites | Frequency |
|---|---|---|---|
| | T0-1 | | |
| | WT | GGGATTAACGATCGATTGCAATGGAAGGAACATTC | 2/9 |
| | d1 | GGGATTAACGATCGATTGCAA-GGAAGGAACATTC | 1/9 |
| | i1 | GGGATTAACGATCGATTGCAATTGGAAGGAACATTC | 6/9 |
| | T0-2 | | |
| | WT | GGGATTAACGATCGATTGCAATGGAAGGAACATTC | 2/13 |
| | d1 | GGGATTAACGATCGATTGCAA-GGAAGGAACATTC | 8/13 |
| | d8 | GGGATTAACGATC--------TGGAAGGAACATTC | 1/13 |
| | d28 | GATCGA--------------------------TTGCAA | 1/13 |
| | i1 | GGGATTAACGATCGATTGCAATTGGAAGGAACATTC | 1/13 |
| | T0-3 | | |
| | WT | GGGATTAACGATCGATTGCAATGGAAGGAACATTC | |
| | | GGGATTAACGATCGATTGCAATGGAAGGAACATTC | |
| | T0-4 | | |
| | WT | GGGATTAACGATCGATTGCAATGGAAGGAACATTC | 3/5 |
| | d28 | GGGATTAACGATCGATTGCAA-------------- | 1/5 |
| | c14 | GGGATTAACGATTGGAAGGAA---------CATTC | 1/5 |
| | T0-5 | | |
| | WT | GGGATTAACGATCGATTGCAATGGAAGGAACATTC | 3/10 |
| | d2 | GGGATTAACGATCGATTGC--TGGAAGGAACATTC | 1/10 |
| | i1 | GGGATTAACGATCGATTGCAATTGGAAGGAACATTC | 6/10 |
| | T0-6 | | |
| | WT | GGGATTAACGATCGATTGCAATGGAAGGAACATTC | 7/10 |
| | d6 | GGGATTAACGATCGAT------GGAAGGAACATTC | 1/10 |
| | i1 | GGGATTAACGATCGATTGCAATTGGAAGGAACATTC | 1/10 |
| sgRNA1 | c5 | GGGATTAACGATCGATTGCAATGAAA-----CATTC | 1/10 |
| *SlPDS-* | T0-7 | | |
| | WT | GGGATTAACGATCGATTGCAATGGAAGGAACATTC | |
| | | GGGATTAACGATCGATTGCAATGGAAGGAACATTC | |
| | T0-8 | | |
| | WT | GGGATTAACGATCGATTGCAATGGAAGGAACATTC | |
| | | GGGATTAACGATCGATTGCAATGGAAGGAACATTC | |
| | T0-9 | | |
| | WT | GGGATTAACGATCGATTGCAATGGAAGGAACATTC | |
| | | GGGATTAACGATCGATTGCAATGGAAGGAACATTC | |
| | T0-10 | | |
| | WT | GGGATTAACGATCGATTGCAATGGAAGGAACATTC | 4/10 |
| | d2 | GGGATTAACGATCGATTGC--TGGAAGGAACATTC | 2/10 |
| | d65 | ATTAGG--------------------------AATTCA | 1/10 |
| | i1 | GGGATTAACGATCGATTGCAATTGGAAGGAACATTC | 3/10 |

| | | | |
|---|---|---|---|
| | T0-11 | | |
| | WT | GGGATTAACGATCGATTGCAATGGAAGGAACATTC | 3/7 |
| | d1 | GGGATTAACGATCGATTGCAA-GGAAGGAACATTC | 1/7 |
| | d1b | GGGATTAACGATCGATTGCA-TGGAAGGAACATTC | 1/7 |
| | i1 | GGGATTAACGATCGATTGCAATTGGAAGGAACATTC | 2/7 |
| | T0-12 | | |
| | WT | GGGATTAACGATCGATTGCAATGGAAGGAACATTC | 3/7 |
| | d3 | GGGATTAACGATCGATTGC---GGAAGGAACATTC | 1/7 |
| | d72 | GGAGAATT------------------------CAGCCG | 3/7 |
| | T0-13 | | |
| | WT | GGGATTAACGATCGATTGCAATGGAAGGAACATTC | |
| | | GGGATTAACGATCGATTGCAATGGAAGGAACATTC | |
| sgRNA1 | T0-13 | Mixed tissues | |
| *SlPDS-* | WT | GGGATTAACGATCGATTGCAATGGAAGGAACATTC | |
| | | GGGATTAACGATCGATTGCAATGGAAGGAACATTC | |
| | T0-14 | | |
| | WT | GGGATTAACGATCGATTGCAATGGAAGGAACATTC | 3/8 |
| | d55 | TATTCA---------------------------ATGATA | 1/8 |
| | i1 | GGGATTAACGATCGATTGCAATTGGAAGGAACATTC | 2/8 |
| | i1b | GGGATTAACGATCGATTGCAAATGGAAGGAACATTC | 2/8 |
| | T0-15 | | |
| | WT | GGGATTAACGATCGATTGCAATGGAAGGAACATTC | 4/6 |
| | d1 | GGGATTAACGATCGATTGCA-TGGAAGGAACATTC | 1/6 |
| | i1 | GGGATTAACGATCGATTGCAATTGGAAGGAACATTC | 1/6 |
| | T0-16 | | |
| | WT | GGGATTAACGATCGATTGCAATGGAAGGAACATTC | |
| | d1 | GGGATTAACGATCGATTGCAA-GGAAGGAACATTC | 3/10 |
| | i1 | GGGATTAACGATCGATTGCAATTGGAAGGAACATTC | 6/10 |
| | i1a | GGGATTAACGATCGATTGCAATAGGAAGGAACATTC | 1/10 |
| | T0-18 | | |
| | WT | GGGATTAACGATCGATTGCAATGGAAGGAACATTC | |
| | | GGGATTAACGATCGATTGCAATGGAAGGAACATTC | |
| | T0-18 | Mixed tissues | |
| | WT | GGGATTAACGATCGATTGCAATGGAAGGAACATTC | |
| | | GGGATTAACGATCGATTGCAATGGAAGGAACATTC | |
| | T0-19 | | |
| | WT | GGGATTAACGATCGATTGCAATGGAAGGAACATTC | 3/11 |
| | i1 | GGGATTAACGATCGATTGCAATTGGAAGGAACATTC | 1/11 |
| | d5 | GGGATTAACGATCGATTG-----GAAGGAACATTC | 5/11 |
| | d12 | GGGATTAACGATCG------------GGAACATTC | 2/11 |
| | T0-20 | | |
| | WT | GGGATTAACGATCGATTGCAATGGAAGGAACATTC | 6/10 |
| | d5 | GGGATTAACGATCGATT-----GGAAGGAACATTC | 2/10 |
| | d12 | GGGATTAACGATCG------------GGAACATTC | 1/10 |

| | | | |
|---|---|---|---|
| | c102 | GCTTAC------------------A----AGCCGC | 1/10 |
| | T0-21 | | |
| | WT | GGGATTAACGATCGATTGCAATGGAAGGAACATTC | |
| | i1 | GGGATTAACGATCGATTGCAATTGGAAGGAACATTC | 2/4 |
| | d2 | GGGATTAACGATCGATTGCAAT--AAGGAACATTC | 1/4 |
| | d4 | GGGATTAACGATCGATTG----GGAAGGAACATTC | 1/4 |
| | T0-22 | | |
| | WT | GGGATTAACGATCGATTGCAATGGAAGGAACATTC | 2/10 |
| | i1 | GGGATTAACGATCGATTGCAATTGGAAGGAACATTC | 7/10 |
| sgRNA1 | d2 | GGGATTAACGATCGATTGC--TGGAAGGAACATTC | 1/10 |
| -*SlPDS* | T0-22 | Mixed tissues | |
| | WT | GGGATTAACGATCGATTGCAATGGAAGGAACATTC | 6/10 |
| | i1 | GGGATTAACGATCGATTGCAATTGGAAGGAACATTC | 2/10 |
| | d6 | GGGATTAACGATCGAT------GGAAGGAACATTC | 1/10 |
| | d13 | GGGATTAACGATC-------------GGAACATTC | 1/10 |
| | T0-1 | | |
| | WT | CAATTGGACTCTTGCCAGCAATGCTTGGAGGGCAA | 1/5 |
| | d4 | CAATTGGACTCTTGCCA----TGCTTGGAGGGCAA | 1/5 |
| | d23 | C-----------------------TTGGAGGGCAA | 1/5 |
| | d2 | CAATTGGACTCTTGCCAGC--TGCTTGGAGGGCAA | 1/5 |
| | i2 | AATTGGACTCTTGCCAGCAATATGCTTGGAGGGCAA | 1/5 |
| | T0-2 | | |
| | WT | CAATTGGACTCTTGCCAGCAATGCTTGGAGGGCAA | |
| | d5 | CAATTGGACTCTTGCC-----TGCTTGGAGGGCAA | 3/5 |
| | d2 | CAATTGGACTCTTGCCAGCA--GCTTGGAGGGCAA | 1/5 |
| | i1 | CAATTGGACTCTTGCCAGCAATTGCTTGGAGGGCAA | 1/5 |
| | T0-3 | | |
| | WT | CAATTGGACTCTTGCCAGCAATGCTTGGAGGGCAA | 1/5 |
| | d1 | CAATTGGACTCTTGCCAGCA-TGCTTGGAGGGCAA | 3/5 |
| | d5 | CAATTGGACTCTTGCC-----TGCTTGGAGGGCAA | 1/5 |
| | T0-4 | | |
| | WT | CAATTGGACTCTTGCCAGCAATGCTTGGAGGGCAA | |
| | d1 | CAATTGGACTCTTGCCAGCA-TGCTTGGAGGGCAA | 2/5 |
| sgRNA2 | d3 | CAATTGGACTCTTGCCAG---TGCTTGGAGGGCAA | 1/5 |
| -*SlPDS* | d4 | CAATTGGACTCTTGCCA----TGCTTGGAGGGCAA | 1/5 |
| | d102 | CTTA------------------------------AAAG | 1/5 |
| | T0-5 | | |
| | WT | CAATTGGACTCTTGCCAGCAATGCTTGGAGGGCAA | |
| | d5 | CAATTGGACTCTTGCCAGC-----TTGGAGGGCAA | 5/19 |
| | d3 | CAATTGGACTCTTGCCAGCAA---TTGGAGGGCAA | 14/19 |
| | T0-6 | | |
| | WT | CAATTGGACTCTTGCCAGCAATGCTTGGAGGGCAA | 3/6 |
| | d3 | CAATTGGACTCTTGCCAGC---GCTTGGAGGGCAA | 2/6 |
| | d1 | CAATTGGACTCTTGCCAGCAA-GCTTGGAGGGCAA | 1/6 |

| | | | |
|---|---|---|---|
| | T0-7 | | |
| | WT | CAATTGGACTCTTGCCAGCAATGCTTGGAGGGCAA | |
| | d14 | CAATTGG--------------TGCTTGGAGGGCAA | 4/11 |
| | d5 | CAATTGGACTCTTGCCA-----GCTTGGAGGGCAA | 1/11 |
| | d3 | CAATTGGACTCTTGCCAGC---GCTTGGAGGGCAA | 6/11 |
| | T0-1 | | |
| | WT | ATGGGAATGGGGATGGGTCCGCCTTCGGTGCCTTC | 2/4 |
| | d6 | ATGGGAATGGGGATGG------CTTCGGTGCCTTC | 1/4 |
| | d1 | ATGGGAATGGGGATGGGTCCG-CTTCGGTGCCTTC | 1/4 |
| | T0-1(1) | | |
| | WT | ATGGGAATGGGGATGGGTCCGCCTTCGGTGCCTTC | 3/9 |
| sgRNA1 | d10 | ATGGGAATGGGGAT----------TCGGTGCCTTC | 4/9 |
| *-SlPIF4* | d1 | ATGGGAATGGGGATGGGTCCG-CTTCGGTGCCTTC | 2/9 |
| | T0-1(2) | | |
| | WT | ATGGGAATGGGGATGGGTCCGCCTTCGGTGCCTTC | 3/10 |
| | d10 | ATGGGAATGGGGAT----------TCGGTGCCTTC | 6/10 |
| | d1 | ATGGGAATGGGGATGGGTCCG-CTTCGGTGCCTTC | 1/10 |
| | T0-2 | | |
| | WT | ATGGGAATGGGGATGGGTCCGCCTTCGGTGCCTTC | 5/9 |
| | d4 | ATGGGAATGGGGATGGGT----CTTCGGTGCCTTC | 2/9 |
| | d18 | ATGG------------------CTTCGGTGCCTTC | 2/9 |
| | T0-3 | | |
| | WT | ATGGGAATGGGGATGGGTCCGCCTTCGGTGCCTTC | |
| | d4 | ATGGGAATGGGGATGGGT----CTTCGGTGCCTTC | 1/9 |
| | i1 | ATGGGAATGGGGATGGGTCCGCACTTCGGTGCCTTC | 5/9 |
| | d3 | ATGGGAATGGGGATGGGTC---CTTCGGTGCCTTC | 3/9 |
| | T0-4 | | |
| | WT | ATGGGAATGGGGATGGGTCCGCCTTCGGTGCCTTC | |
| | d1 | ATGGGAATGGGGATGGGTCC-CCTTCGGTGCCTTC | 2/4 |
| | d20 | ATGGGAA--------------------GTGCCTTC | 1/4 |
| | d61 | CATTT-----------------------------GCTA | 1/4 |
| | T0-5 | | |
| | WT | ATGGGAATGGGGATGGGTCCGCCTTCGGTGCCTTC | 2/4 |
| | d3 | ATGGGAATGGGGATGGGTCCGCC---GGTGCCTTC | 1/4 |
| | d30 | C--------------------------GGTGCCTTC | 1/4 |
| | T0-6 | | |
| | WT | ATGGGAATGGGGATGGGTCCGCCTTCGGTGCCTTC | |
| | | ATGGGAATGGGGATGGGTCCGCCTTCGGTGCCTTC | |
| | T0-6 | Mixed tissues | |
| | WT | ATGGGAATGGGGATGGGTCCGCCTTCGGTGCCTTC | |
| | | ATGGGAATGGGGATGGGTCCGCCTTCGGTGCCTTC | |
| | T0-6(1) | | |
| | WT | ATGGGAATGGGGATGGGTCCGCCTTCGGTGCCTTC | |
| | | ATGGGAATGGGGATGGGTCCGCCTTCGGTGCCTTC | |

| | | | |
|---|---|---|---|
| | T0-6(2) | | |
| | WT | ATGGGAATGGGGATGGGTCCGCCTTCGGTGCCTTC | |
| | | ATGGGAATGGGGATGGGTCCGCCTTCGGTGCCTTC | |
| | T0-7 | | |
| | WT | ATGGGAATGGGGATGGGTCCGCCTTCGGTGCCTTC | |
| | c2 | ATGGGAATGGGGATGGGTCCG-CTTCGGTGCCCTC | 7/12 |
| | c14 | GGGAATGGGGATGGTGTCCAA-CACTACATT-TC | 5/12 |
| | T0-7 | Mixed tissues | |
| sgRNA1 | WT | ATGGGAATGGGGATGGGTCCGCCTTCGGTGCCTTC | |
| -*SlPIF4* | c2 | ATGGGAATGGGGATGGGTCCG-CTTCGGTGCCCTC | 4/10 |
| | c14 | GGGAATGGGGATGGTGTCCAA-CACTACATT-TC | 6/10 |
| | T0-7(1) | | |
| | WT | ATGGGAATGGGGATGGGTCCGCCTTCGGTGCCTTC | |
| | c2 | ATGGGAATGGGGATGGGTCCG-CTTCGGTGCCCTC | 8/11 |
| | c14 | GGGAATGGGGATGGTGTCCAA-CACTACATT-TC | 3/11 |
| | T0-8 | | |
| | WT | ATGGGAATGGGGATGGGTCCGCCTTCGGTGCCTTC | 1/4 |
| | d7 | ATGGGAATGGGGATG-------CTTCGGTGCCTTC | 1/4 |
| | d1 | ATGGGAATGGGGATGGGTCCG-CTTCGGTGCCTTC | 1/4 |
| | d3 | ATGGGAATGGGGATGGGTC---CTTCGGTGCCTTC | 1/4 |
| | T0-9 | | |
| | WT | ATGGGAATGGGGATGGGTCCGCCTTCGGTGCCTTC | |
| | d1 | ATGGGAATGGGGATGGGTCCG-CTTCGGTGCCTTC | 2/5 |
| | d18 | ATGGGAATGGGGATGGGTCC------------GCAC | 2/5 |
| | c62 | GTTTCCAACACTACATGTCCA---AATATATGATTC | 1/5 |
| | T0-10 | | |
| | WT | ATGGGAATGGGGATGGGTCCGCCTTCGGTGCCTTC | 1/4 |
| | c51 | TGGTGATATTCAGATGATGTGGATGGGAAGTG--G | 2/4 |
| | c126 | ATATTCAGATGATGTGGATGGGAAGTG--GCATTT | 1/4 |
| | T0-11 | | |
| | WT | ATGGGAATGGGGATGGGTCCGCCTTCGGTGCCTTC | 3/9 |
| | d8 | ATGGGAATGGGGAT--------CTTCGGTGCCTTC | 4/9 |
| | c52 | TTCCA------TGCACAATGCTAT-----GCATTT | 1/9 |
| | c51 | TGATATTCAGATGATGTGGATGGGAAGTG-GCATT | 1/9 |
| | T0-12 | | |
| | WT | ATGGGAATGGGGATGGGTCCGCCTTCGGTGCCTTC | |
| | | ATGGGAATGGGGATGGGTCCGCCTTCGGTGCCTTC | |
| sgRNA1 | T0-13 | | |
| -*SlPIF4* | WT | ATGGGAATGGGGATGGGTCCGCCTTCGGTGCCTTC | |
| | d1 | ATGGGAATGGGGATGGGTCCG-CTTCGGTGCCTTC | 2/4 |
| | d4 | ATGGGAATGGGGATGGGT----CTTCGGTGCCTTC | 1/4 |
| | d7 | ATGGGAATGGGGATG-------CTTCGGTGCCTTC | 1/4 |
| | T0-14 | | |
| | WT | ATGGGAATGGGGATGGGTCCGCCTTCGGTGCCTTC | 1/5 |

| | | | |
|---|---|---|---|
| | d1 | ATGGGAATGGGGATGGGTCCG-CTTCGGTGCCTTC | 2/5 |
| | d4 | ATGGGAATGGGGATGGGT----CTTCGGTGCCTTC | 2/5 |
| | T0-15 | | |
| | WT | ATGGGAATGGGGATGGGTCCGCCTTCGGTGCCTTC | |
| | d3 | ATGGGAATGGGGATGGGTCCGC---CGGTGCCTTC | 4/12 |
| | d1 | ATGGGAATGGGGATGGGTCCG-CTTCGGTGCCTTC | 8/12 |
| | T0-15 | Mixed tissues | |
| | WT | ATGGGAATGGGGATGGGTCCGCCTTCGGTGCCTTC | |
| | d3 | ATGGGAATGGGGATGGGTCCGC---CGGTGCCTTC | 4/9 |
| | d1 | ATGGGAATGGGGATGGGTCCG-CTTCGGTGCCTTC | 5/9 |
| | T0-16 | | |
| | WT | ATGGGAATGGGGATGGGTCCGCCTTCGGTGCCTTC | |
| | d3 | ATGGGAATGGGGATGGGTCCGC---CGGTGCCTTC | 5/9 |
| | d5 | ATGGGAATGGGGATGGGTC-----TTCGGTGCCTTC | 1/9 |
| | d1 | ATGGGAATGGGGATGGGTCCG-CTTCGGTGCCTTC | 2/9 |
| | i1 | ATGGGAATGGGGATGGGTCCGCACTTCGGTGCCTTC | 1/9 |
| | T0-17 | | |
| | WT | ATGGGAATGGGGATGGGTCCGCCTTCGGTGCCTTC | |
| | | ATGGGAATGGGGATGGGTCCGCCTTCGGTGCCTTC | |
| | T0-18 | | |
| | WT | ATGGGAATGGGGATGGGTCCGCCTTCGGTGCCTTC | |
| | | ATGGGAATGGGGATGGGTCCGCCTTCGGTGCCTTC | |
| | T0-19 | ATGGGAATGGGGATGGGTCCGCCTTCGGTGCCTTC | |
| | d1 | ATGGGAATGGGGATGGGTCCG-CTTCGGTGCCTTC | 9/10 |
| sgRNA1 | d19 | ATGG-------------------TTCGGTGCCTTC | 1/10 |
| -*SlPIF4* | T0-20 | | |
| | WT | ATGGGAATGGGGATGGGTCCGCCTTCGGTGCCTTC | 2/4 |
| | d1 | ATGGGAATGGGGATGGGTCCG-CTTCGGTGCCTTC | 1/4 |
| | d5 | ATGGGAATGGGGATGGG-----CTTCGGTGCCTTC | 1/4 |
| | T0-21 | | |
| | WT | ATGGGAATGGGGATGGGTCCGCCTTCGGTGCCTTC | |
| | i1 | ATGGGAATGGGGATGGGTCCGCACTTCGGTGCCTTC | 3/9 |
| | d1 | ATGGGAATGGGGATGGGTCCG-CTTCGGTGCCTTC | 4/9 |
| | d4 | ATGGGAATGGGGATGGGT----CTTCGGTGCCTTC | 1/9 |
| | d50 | GGTGTC---------------------------TTCC | 1/9 |
| | T0-22 | | |
| | WT | ATGGGAATGGGGATGGGTCCGCCTTCGGTGCCTTC | |
| | d1 | ATGGGAATGGGGATGGGTCCG-CTTCGGTGCCTTC | |
| | T0-22 | Mixed tissues | |
| | WT | ATGGGAATGGGGATGGGTCCGCCTTCGGTGCCTTC | |
| | d1 | ATGGGAATGGGGATGGGTCCG-CTTCGGTGCCTTC | |
| | T0-22(1) | | |
| | WT | ATGGGAATGGGGATGGGTCCGCCTTCGGTGCCTTC | |
| | d1 | ATGGGAATGGGGATGGGTCCG-CTTCGGTGCCTTC | |

| | | | |
|---|---|---|---|
| | T0-22(2) | | |
| | WT | ATGGGAATGGGGATGGGTCCGCCTTCGGTGCCTTC | |
| | d1 | ATGGGAATGGGGATGGGTCCG-CTTCGGTGCCTTC | |
| | T0-23 | | |
| | WT | ATGGGAATGGGGATGGGTCCGCCTTCGGTGCCTTC | |
| | d1 | ATGGGAATGGGGATGGGTCCG-CTTCGGTGCCTTC | |
| | T0-23 | Mixed tissues | |
| | WT | ATGGGAATGGGGATGGGTCCGCCTTCGGTGCCTTC | |
| | d1 | ATGGGAATGGGGATGGGTCCG-CTTCGGTGCCTTC | |
| | T0-24 | | |
| | WT | ATGGGAATGGGGATGGGTCCGCCTTCGGTGCCTTC | 1/4 |
| | d1 | ATGGGAATGGGGATGGGTCCG-CTTCGGTGCCTTC | 2/4 |
| | d4 | ATGGGAATGGGGATGGGT----CTTCGGTGCCTTC | 1/4 |
| | T0-25 | | |
| | WT | ATGGGAATGGGGATGGGTCCGCCTTCGGTGCCTTC | |
| | d1 | ATGGGAATGGGGATGGGTCCG-CTTCGGTGCCTTC | 5/7 |
| sgRNA1 | d5 | ATGGGAATGGGGATGGG-----CTTCGGTGCCTTC | 1/7 |
| -*SlPIF4* | d4 | ATGGGAATGGGGATGGGT----CTTCGGTGCCTTC | 1/7 |
| | T0-1 | | |
| | WT | GTAGAGTGAGGAAGAAGCTCTTGCAAGGCCTTCAT | 3/9 |
| | i1 | GTAGAGTGAGGAAGAAGCTCTTTGCAAGGCCTTCAT | 5/9 |
| | d128 | CATGA----------------------TTAAGGTG | 1/9 |
| | T0-2 | | |
| | WT | GTAGAGTGAGGAAGAAGCTCTTGCAAGGCCTTCAT | |
| | d3 | GTAGAGTGAGGAAGAAGCT---GCAAGGCCTTCAT | 2/15 |
| sgRNA2 | d1 | GTAGAGTGAGGAAGAAGCTCT-GCAAGGCCTTCAT | 13/15 |
| -*SlPIF4* | T0-3 | | |
| | WT | GTAGAGTGAGGAAGAAGCTCTTGCAAGGCCTTCAT | |
| | | GTAGAGTGAGGAAGAAGCTCTTGCAAGGCCTTCAT | |
| | T0-4 | | |
| | WT | GTAGAGTGAGGAAGAAGCTCTTGCAAGGCCTTCAT | |
| | d6 | GTAGAGTGAGGAAGAA------GCAAGGCCTTCAT | 4/8 |
| | d1 | GTAGAGTGAGGAAGAAGCTCT-GCAAGGCCTTCAT | 1/8 |
| | d17 | GTAGAGTGAGG-----------------CCTTCAT | 3/8 |
| | T0-5 | | |
| | WT | GTAGAGTGAGGAAGAAGCTCTTGCAAGGCCTTCAT | |
| | | GTAGAGTGAGGAAGAAGCTCTTGCAAGGCCTTCAT | |
| | T0-5 | Mixed tissues | |
| | WT | GTAGAGTGAGGAAGAAGCTCTTGCAAGGCCTTCAT | |
| | | GTAGAGTGAGGAAGAAGCTCTTGCAAGGCCTTCAT | |
| | T0-5(1) | | |
| | WT | GTAGAGTGAGGAAGAAGCTCTTGCAAGGCCTTCAT | |
| | | GTAGAGTGAGGAAGAAGCTCTTGCAAGGCCTTCAT | |
| | T0-5(2) | | |

| | | | |
|---|---|---|---|
| | WT | GTAGAGTGAGGAAGAAGCTCTTGCAAGGCCTTCAT | |
| | | GTAGAGTGAGGAAGAAGCTCTTGCAAGGCCTTCAT | |
| | T0-6 | | |
| | WT | GTAGAGTGAGGAAGAAGCTCTTGCAAGGCCTTCAT | |
| | d2 | GTAGAGTGAGGAAGAAGCT--TGCAAGGCCTTCAT | 3/10 |
| | d3 | GTAGAGTGAGGAAGAAGCT---GCAAGGCCTTCAT | 2/10 |
| | d9 | GTAGAGTGAGGAA---------GCAAGGCCTTCAT | 2/10 |
| | d2a | GTAGAGTGAGGAAGAAGCTC--GCAAGGCCTTCAT | 2/10 |
| | d4 | GTAGAGTGAGGAAGAAGC----GCAAGGCCTTCAT | 1/10 |
| | T0-7 | | |
| | WT | GTAGAGTGAGGAAGAAGCTCTTGCAAGGCCTTCAT | |
| | d9 | GTAGAGTGAGGAA---------GCAAGGCCTTCAT | 4/13 |
| | d2 | GTAGAGTGAGGAAGAAGCTC--GCAAGGCCTTCAT | 9/13 |
| | T0-7 | Mixed tissues | |
| | WT | GTAGAGTGAGGAAGAAGCTCTTGCAAGGCCTTCAT | |
| | d9 | GTAGAGTGAGGAA---------GCAAGGCCTTCAT | 2/7 |
| | d2 | GTAGAGTGAGGAAGAAGCTC--GCAAGGCCTTCAT | 5/7 |
| | T0-7(1) | | |
| | WT | GTAGAGTGAGGAAGAAGCTCTTGCAAGGCCTTCAT | |
| | d9 | GTAGAGTGAGGAA---------GCAAGGCCTTCAT | 3/9 |
| | d2 | GTAGAGTGAGGAAGAAGCTC--GCAAGGCCTTCAT | 6/9 |
| | T0-7(2) | | |
| | WT | GTAGAGTGAGGAAGAAGCTCTTGCAAGGCCTTCAT | |
| | d9 | GTAGAGTGAGGAA---------GCAAGGCCTTCAT | 4/8 |
| | d2 | GTAGAGTGAGGAAGAAGCTC--GCAAGGCCTTCAT | 4/8 |
| | T0-8 | | |
| | WT | GTAGAGTGAGGAAGAAGCTCTTGCAAGGCCTTCAT | |
| | d2 | GTAGAGTGAGGAAGAAGCTC--GCAAGGCCTTCAT | 6/12 |
| | d9 | GTAGAGTGAGGAA---------GCAAGGCCTTCAT | 6/12 |
| | T0-9 | | |
| | WT | GTAGAGTGAGGAAGAAGCTCTTGCAAGGCCTTCAT | |
| | d6 | GTAGAGTGAGGAAGA------TGCAAGGCCTTCAT | 2/10 |
| | d3 | GTAGAGTGAGGAAGAAGC---TGCAAGGCCTTCAT | 5/10 |
| | d1 | GTAGAGTGAGGAAGAAGCTCT-GCAAGGCCTTCAT | 3/10 |
| | T0-9 | Mixed tissues | |
| | WT | GTAGAGTGAGGAAGAAGCTCTTGCAAGGCCTTCAT | |
| | d6 | GTAGAGTGAGGAAGA------TGCAAGGCCTTCAT | 3/10 |
| sgRNA2 | d3 | GTAGAGTGAGGAAGAAGC---TGCAAGGCCTTCAT | 5/10 |
| *-SlPIF4* | d1 | GTAGAGTGAGGAAGAAGCTCT-GCAAGGCCTTCAT | 2/10 |
| | T0-9(1) | | |
| | WT | GTAGAGTGAGGAAGAAGCTCTTGCAAGGCCTTCAT | 2/10 |
| | d3 | GTAGAGTGAGGAAGAAG---TTGCAAGGCCTTCAT | 2/10 |
| | d2 | GTAGAGTGAGGAAGAAGC--TTGCAAGGCCTTCAT | 1/10 |
| | i1 | GTAGAGTGAGGAAGAAGCTCTTTGCAAGGCCTTCAT | 5/10 |

| | | | |
|---|---|---|---|
| | T0-9(2) | | |
| | WT | GTAGAGTGAGGAAGAAGCTCTTGCAAGGCCTTCAT | 7/9 |
| | i1 | GTAGAGTGAGGAAGAAGCTCTTTGCAAGGCCTTCAT | 1/9 |
| | d1 | GTAGAGTGAGGAAGAAGCTCT-GCAAGGCCTTCAT | 1/9 |
| | T0-10 | | |
| | WT | GTAGAGTGAGGAAGAAGCTCTTGCAAGGCCTTCAT | |
| | d3 | GTAGAGTGAGGAAGAAGCT---GCAAGGCCTTCAT | 6/13 |
| | i1 | GTAGAGTGAGGAAGAAGCTCTTTGCAAGGCCTTCAT | 7/13 |
| | T0-11 | | |
| | WT | GTAGAGTGAGGAAGAAGCTCTTGCAAGGCCTTCAT | |
| | d3 | GTAGAGTGAGGAAGAAGCT---GCAAGGCCTTCAT | 1/6 |
| | i1 | GTAGAGTGAGGAAGAAGCTCTTTGCAAGGCCTTCAT | 2/6 |
| | d2 | GTAGAGTGAGGAAGAAGCT--TGCAAGGCCTTCAT | 3/6 |
| | T0-12 | | |
| | WT | GTAGAGTGAGGAAGAAGCTCTTGCAAGGCCTTCAT | |
| | d17 | GTAGAGTGAGG-----------------CCTTCAT | 1/9 |
| | d6 | GTAGAGTGAGGAAGAA------GCAAGGCCTTCAT | 1/9 |
| | d2 | GTAGAGTGAGGAAGAAGCTC--GCAAGGCCTTCAT | 3/9 |
| sgRNA2 | d6a | GTAGAGTGAGGAAGA------TGCAAGGCCTTCAT | 4/9 |
| -*SlPIF4* | T0-13 | | |
| | WT | GTAGAGTGAGGAAGAAGCTCTTGCAAGGCCTTCAT | |
| | d1 | GTAGAGTGAGGAAGAAGCTCT-GCAAGGCCTTCAT | |
| | T0-13 | Mixed tissues | |
| | WT | GTAGAGTGAGGAAGAAGCTCTTGCAAGGCCTTCAT | |
| | d1 | GTAGAGTGAGGAAGAAGCTCT-GCAAGGCCTTCAT | |
| | T0-13(1) | | |
| | WT | GTAGAGTGAGGAAGAAGCTCTTGCAAGGCCTTCAT | |
| | d1 | GTAGAGTGAGGAAGAAGCTCT-GCAAGGCCTTCAT | |
| | T0-13(2) | | |
| | WT | GTAGAGTGAGGAAGAAGCTCTTGCAAGGCCTTCAT | |
| | d1 | GTAGAGTGAGGAAGAAGCTCT-GCAAGGCCTTCAT | |
| | T0-14 | | |
| | WT | GTAGAGTGAGGAAGAAGCTCTTGCAAGGCCTTCAT | |
| | d17 | GTAGAGTGAGG-----------------CCTTCAT | 1/8 |
| | d6 | GTAGAGTGAGGAAGAA------GCAAGGCCTTCAT | 3/8 |
| | d1 | GTAGAGTGAGGAAGAAGCTCT-GCAAGGCCTTCAT | 2/8 |
| | i1 | GTAGAGTGAGGAAGAAGCTCTTTGCAAGGCCTTCAT | 2/8 |
| | T0-15 | | |
| | WT | GTAGAGTGAGGAAGAAGCTCTTGCAAGGCCTTCAT | |
| | d1 | GTAGAGTGAGGAAGAAGCTCT-GCAAGGCCTTCAT | 5/10 |
| | d2 | GTAGAGTGAGGAAGAAGCT--TGCAAGGCCTTCAT | 4/10 |
| | d2a | GTAGAGTGAGGAAGAAGCTC--GCAAGGCCTTCAT | 1/10 |
| | T0-15 | Mixed tissues | |
| | WT | GTAGAGTGAGGAAGAAGCTCTTGCAAGGCCTTCAT | |

| | | | |
|---|---|---|---|
| | d1 | GTAGAGTGAGGAAGAAGCTCT-GCAAGGCCTTCAT | 8/16 |
| | d2 | GTAGAGTGAGGAAGAAGCT--TGCAAGGCCTTCAT | 8/16 |
| | T0-16 | | |
| | WT | GTAGAGTGAGGAAGAAGCTCTTGCAAGGCCTTCAT | |
| | d1 | GTAGAGTGAGGAAGAAGCTCT-GCAAGGCCTTCAT | 3/5 |
| | d3 | GTAGAGTGAGGAAGAAGCT---GCAAGGCCTTCAT | 1/5 |
| | d2 | GTAGAGTGAGGAAGAAGCTC--GCAAGGCCTTCAT | 1/5 |
| | T0-17 | | |
| | WT | GTAGAGTGAGGAAGAAGCTCTTGCAAGGCCTTCAT | |
| | d1 | GTAGAGTGAGGAAGAAGCTCT-GCAAGGCCTTCAT | |
| | T0-17 | Mixed tissues | |
| | WT | GTAGAGTGAGGAAGAAGCTCTTGCAAGGCCTTCAT | |
| | d1 | GTAGAGTGAGGAAGAAGCTCT-GCAAGGCCTTCAT | |
| | T0-17(1) | | |
| | WT | GTAGAGTGAGGAAGAAGCTCTTGCAAGGCCTTCAT | |
| | d1 | GTAGAGTGAGGAAGAAGCTCT-GCAAGGCCTTCAT | |
| | T0-17(2) | | |
| | WT | GTAGAGTGAGGAAGAAGCTCTTGCAAGGCCTTCAT | |
| | d1 | GTAGAGTGAGGAAGAAGCTCT-GCAAGGCCTTCAT | |
| sgRNA2 | T0-18 | | |
| *-SlPIF4* | WT | GTAGAGTGAGGAAGAAGCTCTTGCAAGGCCTTCAT | |
| | i1 | GTAGAGTGAGGAAGAAGCTCTTTGCAAGGCCTTCAT | 7/13 |
| | c43 | AA--GATCGTGTATACTTTCCGTGCAAGGCCTTCAT | 6/13 |
| | T0-19 | | |
| | WT | GTAGAGTGAGGAAGAAGCTCTTGCAAGGCCTTCAT | |
| | d1 | GTAGAGTGAGGAAGAAGCTCT-GCAAGGCCTTCAT | |
| | T0-19 | Mixed tissues | |
| | WT | GTAGAGTGAGGAAGAAGCTCTTGCAAGGCCTTCAT | |
| | d1 | GTAGAGTGAGGAAGAAGCTCT-GCAAGGCCTTCAT | |

DNA was extracted from the leaves or mixed tissues of T0 plants, then amplified by PCR and sequenced. On the first round, homozygote and WT were identified, with PCR product was one type. For others, DNA were cloned and a number of clones were sequenced. In X/Y, Y is the number clones sequenced, and X is the number of clones which showed a specific sequence. #WT, no mutations were detected at target sites. $ Mixed tissues included leaves, shoots and flowers. d#, # of bp deleted at the target sites; d#a, same number base of deletion at one site; i#, # number of bases insertion at target sites; i#a, same number base of deletion at one site; i#b, same number base of deletion at other sites ; c#: combined mutation. PAM (NGG) is indicated in orange; The 20 bp target sequence is marked in green; the bases colored red and blue mean insertion and substitution respectively.

**Supplementary Table S9.** Chromosomal locations of tomato U6 genes.

| Gene | Chromosome | Strand | Location |
|---|---|---|---|
| *SlU6-1* | ch01 | - | 5813051-5813153 |
| *SlU6-2* | ch01 | + | 92151263-92151365 |
| *SlU6-3* | ch07 | - | 292992-293094 |
| *SlU6-4* | ch07 | + | 327864-327966 |
| *SlU6-5* | ch07 | + | 329337-329439 |
| *SlU6-6* | ch07 | - | 2045653-2045755 |
| *SlU6-7* | ch09 | - | 5169327-5169429 |

**Supplementary Table S10.** Primers used in the study.

| Primer names | Sequence of primers | Aims |
|---|---|---|
| sgRNA1-*SlPDS*-F | 5' TCTCCTATCCTCGTTTGTCGT 3' | Genotyping |
| sgRNA1-*SlPDS*-R | 5' TTGCTTTCTCATCCAGTCCTT 3' | |
| sgRNA2-*SlPDS*-F | 5' ATGGTGAGCTAATCACGAGTAA 3' | |
| sgRNA2-*SlPDS*-R | 5' AAAGAGAGGCAGGTAGACAATC 3' | |
| sgRNA1-*SlPIF4*-F | 5' ATACGGGGTGAGTTAGCGA 3' | |
| sgRNA1-*SlPIF4*-R | 5' AGTGGCAGGTGCATTACTATTA 3' | |
| sgRNA2-*SlPIF4*-F | 5' GAGGAAACAAGTCAGCCCA 3' | |
| sgRNA2-*SlPIF4*-R | 5' TCGCTAACTCACCCCGTAT 3' | |
| OFF1-F | 5' ACGATTGATGGATTGTGAAG 3' | Off-target analysis |
| OFF1-R | 5' TTCAGGGACGAATGTTTGTAG 3' | |
| OFF2-F | 5' GTGCCTTGCCTATCTACTGG 3' | |
| OFF2-R | 5' CATTGCGTCAACATCACCT 3' | |
| OFF3-F | 5' CATGATTAGGCCAGGATTTC 3' | |
| OFF3-R | 5' GAAGTGTAACCCCCCTGAAT 3' | |
| OFF4-F | 5' ACAATGGATTTCACGACTCTC 3' | |
| OFF4-R | 5' AAACCCGTTCCGTCTGTAG 3' | |
| OFF5-F | 5' AGCTGAATATCATAGGTTTTCCT 3' | |
| OFF5-R | 5' CTCTATCACCGAAGTGGCTAG 3' | |
| OFF6-F | 5' AAGAAATCTGTTGTTCAGTATCAGT 3' | |
| OFF6-R | 5' AGTTGTAACGGTTAAAGGGC 3' | |
| OFF7-F | 5' CACCTCAGTTTTCACATCAGC 3' | |
| OFF7-R | 5' CTCGCTCTACGATAGCACATT 3' | |
| OFF8-F | 5' ATGGGACGATAATGAACACTG 3' | |
| OFF8-R | 5' GCATCTCTTTAGGTCAGCATC 3' | |
| OFF9-F | 5' GCCTCCTTACATTACAAGAAACTA 3' | |
| OFF9-R | 5' AGACGCACAAATCGGACTAT 3' | |
| SlUbi-1F | 5' TCCATCTCGTGCTCCGTCT 3' | qRT-PCR of *Cas9* and sgRNA |
| SlUbi-1R | 5' CTGAACCTTTCCAGTGTCAT 3' | |
| Cas9-F | 5' GACGCTAATCTGGACAAAGTG 3' | |
| Cas9-R | 5' TCAGGGTAAACAGGTGGATG 3' | |
| sgRNA1-*SlPDS*-F | 5' CGATTGCAATGGAGTTTTAGAG 3' | |
| sgRNA1-*SlPDS*-R | 5' GTGCCACTTTTTCAAGTTGAT 3' | |
| sgRNA1-*SlPIF4*-F | 5' GGGTCCGCCTTGTTTTAGA 3' | |
| sgRNA1-*SlPIF4*-R | 5' CCGACTCGGTGCCACTT 3' | |
| sgRNA2-*SlPIF4*-R | 5' GAAGCTCTTGCAGTTTTAGAGC 3' | |
| sgRNA2-*SlPIF4*-R | 5' CGGTGCCACTTTTTCAAGT 3' | |
| Cas9-pCAMBIA1301-F | 5' ATAAGCCCATCAGAGAGCAG 3' | Detect the T-DNA region |
| Cas9-pCAMBIA1301-R | 5' TAGCATTCGCCATTCAGG 3' | |
